# Radioiodine Therapy-Induced Conversion of Toxic Adenoma to Graves’ Disease

**DOI:** 10.7759/cureus.8683

**Published:** 2020-06-18

**Authors:** Anis Rehman, Silvana Obici, Abid Yaqub

**Affiliations:** 1 Endocrinology, Southern Illinois University School of Medicine, Springfield, USA; 2 Division of Endocrinology and Metabolism, Stony Brook University, Stony Brook, USA; 3 Division of Endocrinology, Diabetes and Metabolism, University of Cincinnati Medical Center, Cincinnati, USA

**Keywords:** i-131 radioiodine treatment, graves’ disease, toxic nodular disease, toxic adenoma

## Abstract

We present a 50-year-old female who was evaluated for the symptoms of thyrotoxicosis. She had low thyroid stimulating hormone (TSH) 0.02 with normal free thyroxine (FT4) 1.00 (0.61-1.76 ng/dL) and normal total triiodothyronine (TT3) 1.0 (0.60-2.20 ng/mL) levels. Her thyrotropin receptor antibody (TRAbs) and thyroid peroxidase antibody (TPOAb) titers were negative. Thyroid ultrasound revealed an ill-defined, heterogeneous, 1.8 cm x 0.8 cm x 0.7 cm nodule in the left lower lobe. 123-radioiodine (RAI) thyroid scan revealed 38.5% uptake, which was concentrated in the lower left thyroid lobe, a finding consistent with a solitary toxic adenoma of the thyroid.

The patient became clinically and biochemically euthyroid on methimazole (MMI). She then underwent 131-RAI therapy with 12 mCi, which cured her hyperthyroidism with normalization of TSH levels for four months. She then developed overt thyrotoxicosis with low TSH of 0.02, elevated TT3 of 3.2, and normal FT4 of 0.91. Repeat TRAbs and TPOAb were elevated along with diffusely increased uptake on the I-123 RAI thyroid uptake scan, consistent with Graves’ disease (GD). The patient was then placed on MMI again to bridge to definitive treatment with total thyroidectomy. Our case is a rare case where the patient with solitary toxic adenoma with negative TPOAb serology developed GD following I-131 RAI treatment.

## Introduction

The pathogenesis of toxic adenoma (TA) and Graves’ disease (GD) is very distinct. TA results from somatic mutations leading to nodules with autonomous activity and growth [1]. It is more prevalent in older population. On the contrary, GD is more common among the younger population. It is induced by circulating antibodies directed against the thyroid stimulating hormone (TSH) receptor, a G-protein-coupled receptor that stimulates growth and stimulates biosynthesis and release of thyroid hormones [2]. Both TA and GD can present with overt or subclinical thyrotoxicosis.

Graves’ disease commonly presents with signs and symptoms of tachycardia, weight loss, tremors, anxiety, diarrhea, and heat intolerance. Patients may also develop Graves’ ophthalmopathy and dermopathy [3]. Its incidence has been found to increase with a genetic predisposition, particularly with human leukocyte antigen DR3 (HLA DR3), which is associated with an increased incidence of autoimmune processes [3-4]. Interestingly, GD has also been known to be triggered by viral or bacterial infections [4]. Upon review of literature, several case studies have described the onset of GD following I-131 radioiodine (RAI) treatment in toxic nodular goiter [5-12].

I-131 RAI therapy has thyroid-selective destructive properties, which makes it an effective treatment for toxic nodular goiter as well as GD [1]. However, I-131 RAI may lead to the complete destruction of the thyroid gland, resulting in hypothyroidism. Transient hyperthyroidism within zero to eight weeks after I-131 RAI treatment may occur due to radiation thyroiditis. I-131 RAI treatment has been reported to trigger autoimmunity in 5%-5.4% of patients with multinodular goiter and in 0%-5.3% of patients with solitary nodular thyroid adenoma [13]. The incidence of seroconversion to positive titers for thyrotropin receptor antibody (TRAbs) after I-131 RAI therapy has been reported to be 5% [8]. Those with positive thyroid peroxidase antibody (TPOAb) titers before RAI-131 therapy have a much higher risk of seroconversion, which is reported to be 22% in one case series [6, 8].

Here, we present a rare case of serologically TPOAb negative solitary toxic nodule which turned into serologically TPOAb and TRAbs positive GD after I-131 RAI treatment. We also review the medical literature regarding the role of I-131 RAI therapy in triggering an autoimmune response leading to the development of GD in patients with pre-existing nodular goiter.

## Case presentation

A 50-year-old female was referred to our endocrinology clinic with subacute onset of fatigue, palpitations, hot flashes, loose stools, dry skin, tremors, anxiety, and insomnia. There was no prior radiation exposure to head and neck, family history of thyroid or autoimmune disease, or recent exposure to iodinated contrast. She also denied taking any iodine or thyroid supplements. Her physical examination was unremarkable with no clinically palpable thyroid enlargement, Graves’ ophthalmopathy, or dermopathy. She was noted to have slight tremors of outstretched fingers.

Thyroid function tests revealed a TSH low at 0.02 (0.34-5.60 uIU/mL) with normal free thyroxine (FT4) 1.00 (0.61-1.76 ng/dL), normal total triiodothyronine (TT3) 1.1 (0.60-2.20 ng/mL), and normal free triiodothyonine (FT3) of 3.1 (2.0-3.6 pg/mL). Her serology titers were negative for both TRAbs < 0.9 IU/L and TPOAb < 10 IU/mL (see Table 1).

**Figure 1:**
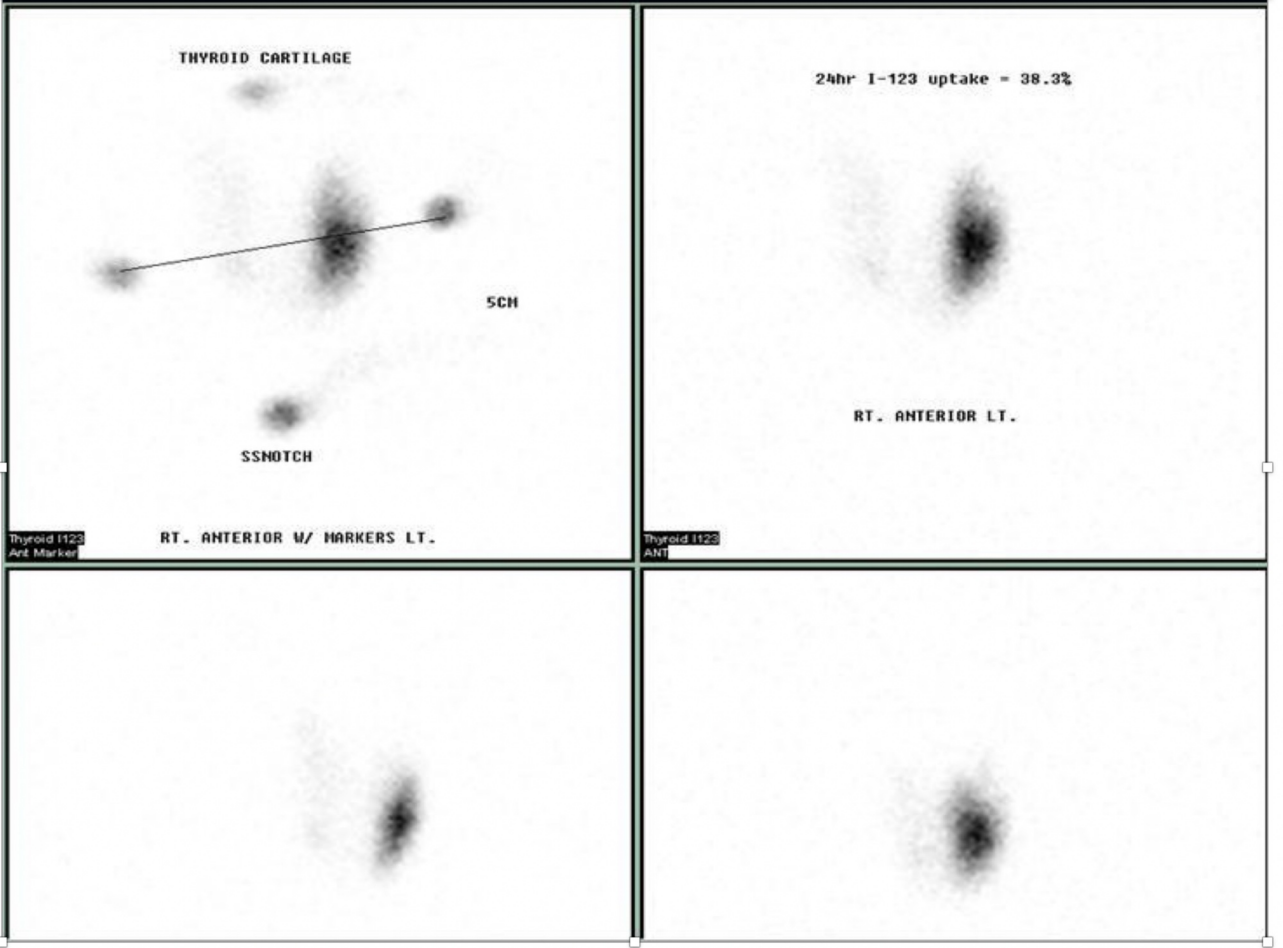
123-RAI thyroid uptake (38.5%) prior to 131-RAI treatment. The report suggests the presence of left lower lobe hot nodule as noted above. RAI, radioiodine

I-123 RAI thyroid scan revealed 38.5% uptake concentrated in the lower portion of the left thyroid lobe, suggesting the presence of a hot nodule in the left lower thyroid lobe consistent with a clinical diagnosis of toxic adenoma (Figure 1). We then evaluated the patient with thyroid ultrasound, which revealed a normal-sized thyroid gland without any hyper-vascularity on color Doppler flow. However, the ultrasound did show an ill-defined, heterogeneous, isoechoic to a hypoechoic nodule, measuring 1.8 cm x 0.8 cm x 0.7 cm in size, which was present in the lower aspect of the left lobe corresponding to the hot area on the thyroid scan. The nodule did not have high-risk features such as micro-calcifications, irregular borders, or being taller than wider in shape.

The patient was started on 5 mg of methimazole (MMI) orally per day, which led to both biochemical and clinical euthyroidism. Her thyroid function test, four months after the initiation of MMI revealed TSH of 0.99 (0.34-5.60 uIU/mL), FT4 of 0.91 (0.61-1.76 ng/dL), TT3 of 1.0 (0.60-2.20 ng/mL), and FT3 of 3.2 (2.0-3.6 pg/mL) (Table 1).

**Table 1:** Timeline for the thyroid function tests. Normal value ranges are as follows: TSH 0.34-5.60 uIU/mL, FT4 0.61-1.76 ng/dL, TT3 0.60-2.20 ng/mL, FT3 2.0-3.6 pg/mL, TRAbs < 0.9 IU/L, and TPOAb < 10 IU/mL. MMI, methimazole; RAI, radioiodine; TSH, thyroid stimulating hormone; FT4, free thyroxine; TT3, total triiodothyronine; FT3, free triiodothyonine; TRAbs, thyrotropin receptor antibody; TPOAb, thyroid peroxidase antibody

Timeline (months)	Events	TSH	FT4	TT3	FT3	TRAbs	TPOAb
0	Pre-MMI and I-131 RAI treatment	0.02 (Low)	1.00	1.1	3.1	<0.9	<10
4	4 months post MMI but pre-I-131 treatment	0.99	0.91	1.0	3.2	NA	NA
5	1 months post I-131 treatment	0.61	0.96	NA	NA	NA	NA
8	4 months post I-131 treatment	0.02 (Low)	1.63	NA	4.6 (High)	10.97 (High)	258 (High)
9	Post total thyroidectomy on levothyroxine 100 mcg/day	1.09	1.00	NA	NA	NA	NA

Upon detailed discussion regarding the risk and benefits of definitive treatment options for hyperthyroidism, the patient decided to proceed with I-131 RAI treatment. A dose of 12 mCi of RAI-131 was given to the patient. A four week follow up visit post I-131 RAI treatment showed TSH of 0.61 (0.34-5.60 uIU/mL), and FT4 of 0.96 (0.61-1.76 ng/dL), which confirmed that the patient was clinically and biochemically euthyroid.

Four months after the I-131 RAI treatment, the patient presented again to our clinic with the onset of palpitations. The repeat blood work revealed low TSH of 0.02 (0.34-5.60 uIU/mL), FT4 levels of 1.63 (0.61-1.76 ng/dL), and elevated FT3 of 4.6 (2.0-3.6 pg/mL), suggesting a relapse of thyrotoxicosis (Table 1).

**Figure 2:**
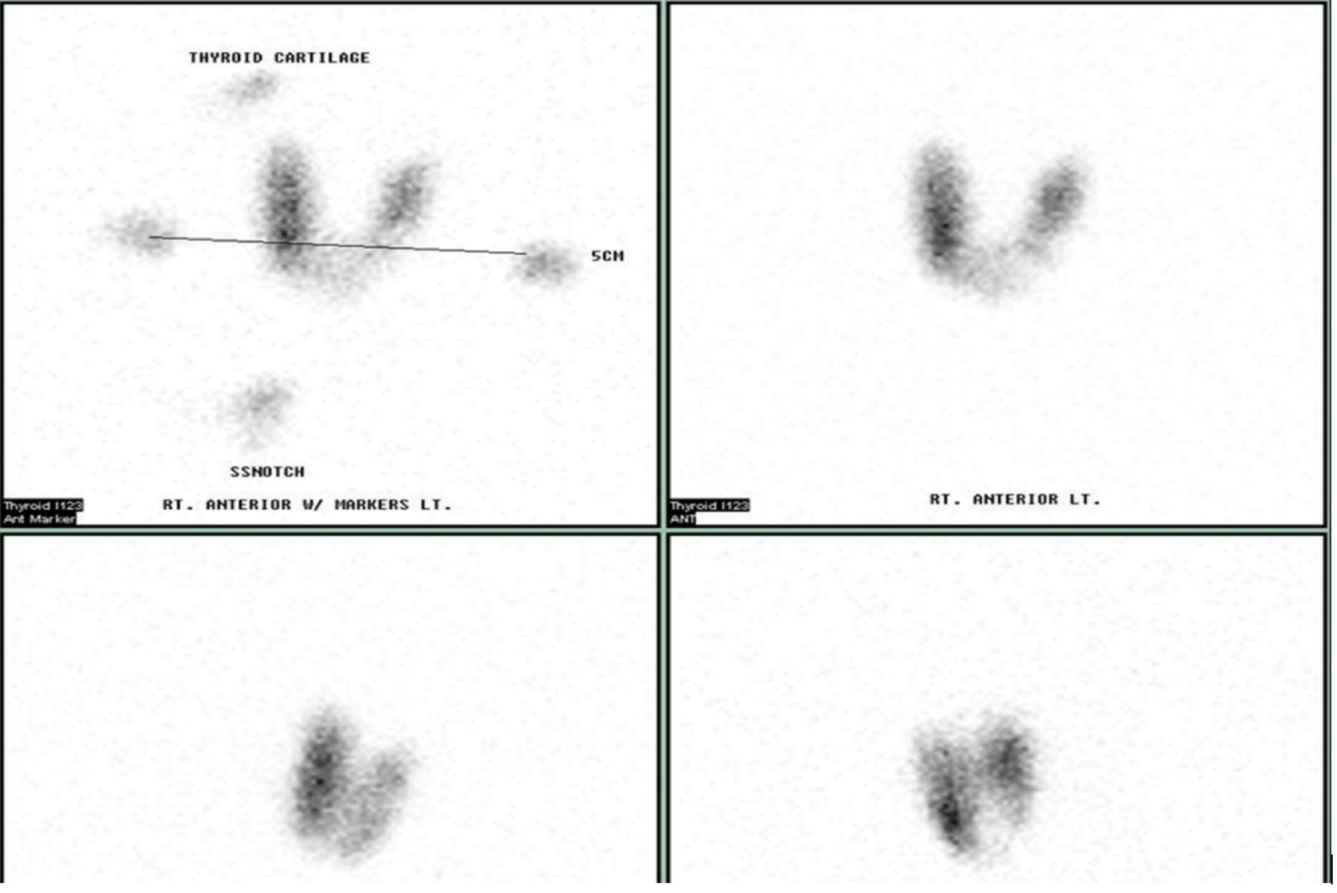
The repeat I-123 RAI scan after I-131 RAI treatment. The figure reveals a diffuse uptake (37.7%) without any localized hot or cold areas/nodules. RAI, radioiodine

Thyrotropin receptor antibody titers were elevated at 10.97 (0.00-1.75 IU/L) and TPOAb titers elevated at 258 (0.0-34.9 IU/mL). The repeat I-123 RAI scan revealed 37.7% diffuse uptake without any localized hot or cold areas/nodules, as noted in Figure 2. The patient was diagnosed with GD, which was likely triggered by the I-131 RAI treatment. We started the patient on MMI, and subsequently, she underwent total thyroidectomy. After the surgery, she was started on levothyroxine and became euthyroid at a dose of 100 mcg daily.

## Discussion

We describe a case of I-131 RAI treatment causing the transition of the toxic nodular disease to GD. It is a rare occurrence, initially described by Boddenberg et al. in 1993 [12], and subsequently reported in a few case reports and small prospective and retrospective studies [5-12]. Besides 1-131 RAI treatment, other conditions that may lead to damage to thyroid cells include surgical manipulation for parathyroidectomy, external radiation therapy for nonthyroidal diseases, subacute thyroiditis, and percutaneous ethanol injections, which have been reported to cause autoimmunity [5, 10]. Interestingly, our patient did not undergo any of these procedures.

**Figure 3:**
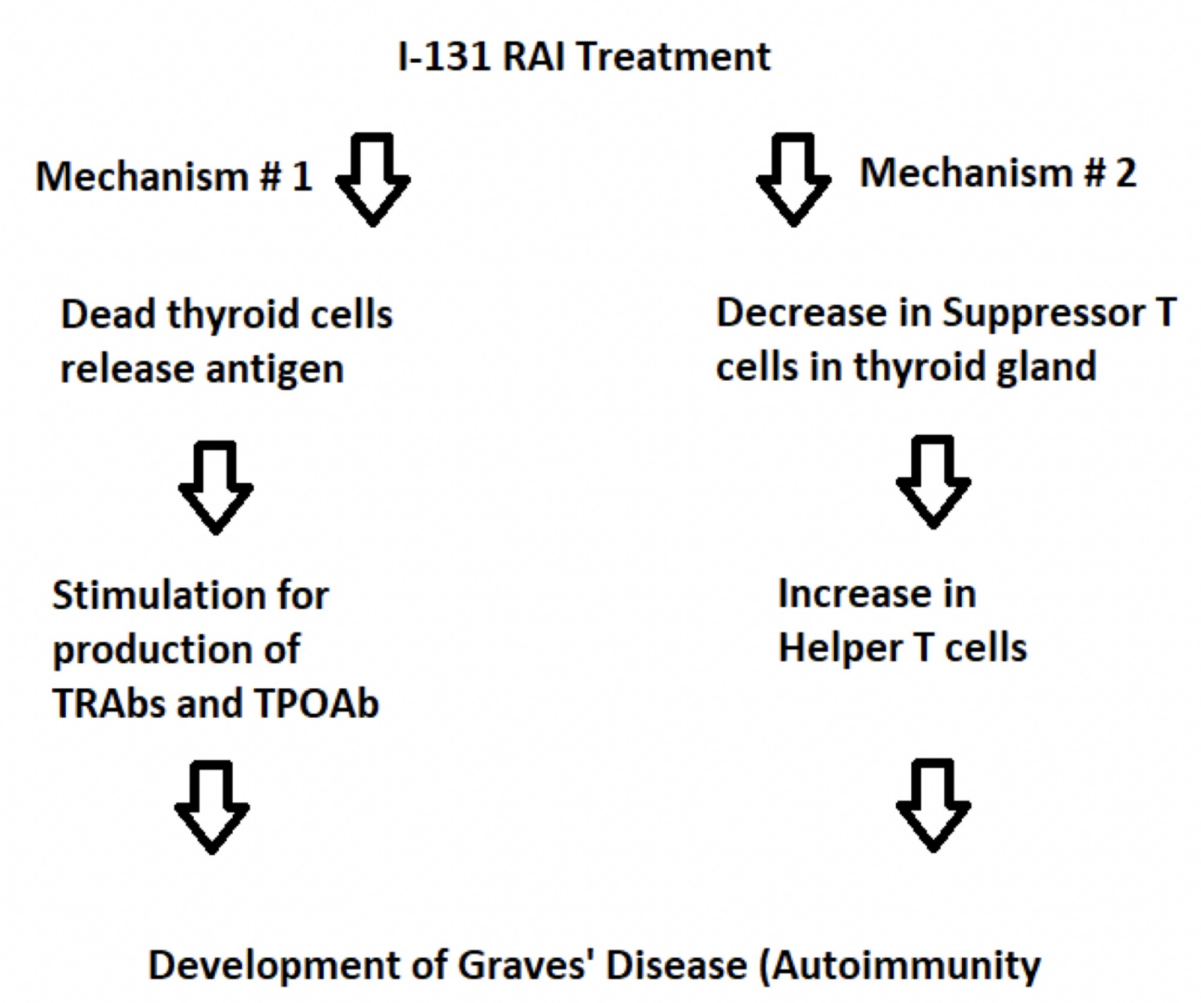
Proposed mechanisms for development of GD after I-131 RAI treatment in toxic nodular disease. GD, Grave’s disease; RAI, radioiodine

A few potential mechanisms have been proposed in the literature to explain I-131 RAI treatment leading to the development of autoimmunity. However, none of them have been specifically tested or proven. One such mechanism is thought to occur in a two-step process with an initial phase of necrosis of thyroid cells, causing the release of antigens, which in turn stimulates autoimmunity against thyroid and production of TSH-stimulating immunoglobulins [5]. NyGaard et al. investigated this concept by postulating that an increase in circulating thyroglobulin (tg) could act as a trigger to autoimmunity after I-131 RAI therapy; however, tg levels were similar in the control group when compared to the patient developing GD after I-131 RAI treatment [5]. It is possible that there is some other antigen, which is the culprit rather than tg. An alternative hypothesis is that I-131 RAI therapy may kill the suppressor T cells in the thyroid, which in turn may cause an imbalance between T helper and suppressor cells resulting in autoimmunity (Figure 3) [11].

A recent systematic review revealed that positive TPOAb titers, glandular hypo-echogenicity, and I-123 RAI diffuse uptake scan, increase risk of development of autoimmunity up to 10-folds after I-131 RAI treatment [10, 12]. Interestingly, in our patient, none of these risk factors were present. Therefore our case is somewhat unusual and unique as the estimated time to onset of autoimmunity after I-131 RAI exposure has been described to be 3-10 months [10-11, 13-14]. In our patient, the timeline was four months post I-131 RAI therapy for the transition from TA to GD. Susceptible genetic make-up along with environmental risk factors is known to contribute to the onset of GD. However our patient denied prior known family history of autoimmune disease [2].

In 1911, Marine and Lenhart reported the presence of thyroid nodules and autoimmunity concomitantly [15]. I-131 RAI therapy leading to the development of autoimmunity is different than Marine-Lenhart syndrome as in the former thyroid nodular disease precedes the development of GD following I-131 RAI treatment [8, 11].

Once diagnosed with hyperthyroidism after more than three months of I-131 RAI treatment, the patients described in the literature have increased uptake on repeat I-123 RAI scan. These patients have positive TPOAb and TRAbs titers, thus confirming GD [5, 11, 14]. Our patient was similar to the cases described in the literature in this regard; she developed positive serology with diffuse uptake on the I-123 RAI scan, a classical presentation of GD, suggesting that I-131 RAI can trigger a de novo autoimmune response. Conversely, not all patients treated with I-131 RAI therapy show seroconversion from negative to positive titers for TPOAb and TRAbs, which suggests that only a subset of predisposed individuals will have this response [5, 14].

Elevated TRAbs titers are detected in more than 90% of patients with GD [2]. However, a small portion of patients has negative autoimmune serology, which could be ascribed to the detection limits of our current serological assays. It has been proposed in the literature that, in some cases, the production of TRAbs titers can be restricted to intra-thyroidal tissue [16]. As a variable degree of lymphocytes infiltration is present in GD, we speculate that our TRAbs-negative patient may have had intra-thyroidal lymphocyte infiltration restricted to only a portion of the thyroid, and thus presenting with I-123 RAI uptake scan typical of the toxic nodule [2-3]. In this scenario, the subsequent I-131 RAI therapy could have triggered exacerbation of an autoimmune reaction against the whole thyroid and the conversion from seronegative to seropositive TRAbs titers.

## Conclusions

Hyperthyroid patients who have nodular thyroid disease with no prior autoimmune disease, and who receive I-131 RAI treatment should be counseled and monitored for GD after 3-10 months of RAI treatment. Positive TPOAb titers, glandular hypo-echogenicity, and diffuse I-123 RAI uptake scan increase the risk of development of autoimmunity. However, it is essential to know that a few patients may still transition to GD from toxic nodular disease after I-131 RAI treatment despite the absence of these risk factors. Although some experts have proposed potential explanations for this phenomenon, the underlying pathogenetic mechanism remains elusive to a large extent. More research is needed in this area to clearly understand the pathophysiologic changes that lead to the development of GD following RAI-131 treatment in patients with nodular thyroid disease.
